# Genome-Wide Identification and Expression Pattern Analysis of KNOX Gene Family in Orchidaceae

**DOI:** 10.3389/fpls.2022.901089

**Published:** 2022-05-27

**Authors:** Diyang Zhang, Siren Lan, Wei-Lun Yin, Zhong-Jian Liu

**Affiliations:** ^1^Key Laboratory of National Forestry and Grassland Administration for Orchid Conservation and Utilization at College of Landscape Architecture, Fujian Agriculture and Forestry University, Fuzhou, China; ^2^College of Biological Sciences and Technology, Beijing Forestry University, Beijing, China

**Keywords:** Orchidaceae, KNOX gene family, stem apical meristem, organ shape, expression pattern

## Abstract

The establishment of lateral organs and subsequent plant architecture involves factors intrinsic to the stem apical meristem (SAM) from which they are derived. *KNOTTED1-LIKE HOMEOBOX* (*KNOX*) genes are a family of plant-specific homeobox transcription factors that especially act in determining stem cell fate in SAM. Although KNOXs have been studied in many land plants for decades, there is a dearth of knowledge on KNOX’s role in Orchidaceae, the largest and most diverse lineage of flowering plants. In this study, a total of 32 putative KNOX genes were identified in the genomes of five orchid species and further designated into two classes (Class I and Class II) based on phylogenetic relationships. Sequence analysis showed that most orchid KNOX proteins retain four conserved domains (KNOX1, KNOX2, ELK, and Homeobox_KN). Comparative analysis of gene structure showed that the exon–intron structure is conserved in the same clade but most orchids exhibited longer intron, which may be a unique feature of Orchidaceae. *Cis*-elements identified in the promoter region of orchid KNOXs were found mostly enriched in a function of light responsiveness, followed by MeJA and ABA responsiveness, indicative of their roles in modulating light and phytohormones. Collinear analysis unraveled a one-to-one correspondence among KNOXs in orchids, and all KNOX genes experienced strong purifying selection, indicating the conservation of this gene family has been reinforced across the Orchidaceae lineage. Expression profiles based on transcriptomic data and real-time reverse transcription–quantitative PCR (RT-qPCR) revealed a stem-specific expression of KNOX Class I genes and a broader expression pattern of Class II genes. Taken together, our results provided a comprehensive analysis to uncover the underlying function of KNOX genes in Orchidaceae.

## Introduction

Homeoproteins identified in almost all eukaryotic lineages are categorized into two superclasses, Three Amino Acid Length Extension (TALE) and non-TALE (Lee et al., 2008). *KNOTTED1-like homeobox* (*KNOX*) genes are one of the TALE superclasses in plants, encoding transcription factors that regulate stem-cell specification, particularly at shoot apical meristem (SAM). KNOX proteins are characterized by four conserved domains: TALE-type homeodomain (Homeobox_KN) at C-terminal; MEINOX domain includes KNOX1 and KNOX2 at N-terminal; and an ELK domain located upstream of homeodomain (Meng et al., 2020). KNOX1 and KNOX2 domains have been previously shown to participate in protein–protein interactions (Reiser et al., 2000). The ELK domain could act as a nuclear localization signal for transcriptional repression, and the homeodomain may function in the recognition of promoter sequences in downstream genes (Bueno et al., 2020). On the basis of the homeodomain similarity, intron, expression patterns, and phylogeny, this small gene family is split into three classes: Class I, Class II, and a newly discovered Class M (Magnani and Hake, 2008). Representatives of Class I and Class II genes are present in bryophytes and all flowering plants, which exhibit conserved but opposing roles (Frangedakis et al., 2017).

Recent studies have shed new light on the function of KNOX genes in several plants including gymnosperms such as *Pinus pinaster* (Bueno et al., 2020), monocots such as rice (Chen et al., 2021) and *Lilium tsingtauense* (Zhou et al., 2022), dicots such as *Malus pumila* (Jia et al., 2021a), while extensive research has only been done in *Arabidopsis thaliana* (Furumizu et al., 2015; Qin et al., 2020). In *A. thaliana*, Class I subfamily consists of four members, *SHOOT MERISTEMLESS* (*STM*), *KNAT1*, *KNAT2*, and *KNAT6* (Hake et al., 2004). These genes are specifically expressed in SAM, which harbors pluripotent stem cells to maintain an indeterminate state but switched off in determinate lateral organs. *STM* functions in shoot apical meristem, maintaining floral meristem and carpel formation with *stm* mutants fail to specify and maintain a SAM (Scofield et al., 2007). *KNAT1*, *KNAT2*, and *KNAT6* act redundantly with *STM* in concert to regulate stem cell maintenance, carpel formation, and embryonic SAM boundaries, respectively (Hay and Tsiantis, 2009). The loss of function of these genes results in irregular floral structure, shortened internodes, and reduced apical dominance (Venglat et al., 2002). Class II KNOX genes include *KNAT3*, *KNAT4*, *KNAT5*, and *KNAT7*, by contrast, have a broader expression in differentiating and mature tissues. Genetic analyses demonstrate that Class II KNOX genes promote differentiation of all aerial organs whilst suppressing meristematic capability, suggesting that they act antagonistically with Class I genes, but their functions remain largely unknown due to the paucity of studies and extensive genetic redundancy (Furumizu et al., 2015; Wang et al., 2020). *KNATM*, the only member of Class M found in some eudicots (Jie et al., 2015), is featured by the absence of ELK–homeodomain region and has a function in leaf proximal–distal patterning (Magnani and Hake, 2008).

Organ initiation from meristems under differing regulating mechanisms could give rise to dramatically distinct morphologies and KNOX genes, with no doubt having essential roles in these processes (Hay and Tsiantis, 2010; Sluis and Hake, 2015). With approximately 750 genera and 28,000 species, Orchidaceae represents one of the largest, most widespread, and species-rich families of angiosperm lineages (Chase et al., 2015). Orchids are also one of the most prestigious, horticulturally significant plants owing to their unique morphology and diversity. Despite extensive studies of KNOX genes that have been done within model plants, little is known about the features of KNOX genes in Orchidaceae. As high-quality, chromosomal-level orchid genomes have emerged recently, the opportunity arises to conduct the systematic study of the orchid KNOX family and investigate its expression in meristems during development and its association with plant architecture. In this study, we perform genome-wide identification, comparative and expression analyses of KNOX genes in five orchids, *Apostasia shenzhenica* (Apostasioideae, the primitive subfamily of Orchidaceae), *Phalaenopsis equestris*, *Cymbidium ensifolium*, *Cymbidium goeringii*, and *Dendrobium chrysotoxum* (Epidendroideae, the advanced subfamily with most diverse species) to illustrate the characteristics of KNOX genes during the evolution of orchids. The results could provide novel insights into the fundamental mechanisms underlying the organ morphology evolution and diversification of orchids and other flowering plants.

## Materials and Methods

### Data Sources

Genome sequences, annotation files, and raw data of transcriptome from different tissues of *A. shenzhenica* (accession number: PRJNA310678), *P. equestris* (accession number: PRJNA53913), *C. goeringii* (accession number: PRJNA749652), and *D. chrysotoxum* (accession number: PRJNA664445) were downloaded from the National Center for Biotechnology Information (NCBI), and data for *C. ensifolium* (accession number: PRJCA005355) was downloaded from the National Genomics Data Center (NGDC).^1^ KNOX proteins of *A. thaliana* and *Oryza sativa* were retrieved from TAIR^2^ and Phytozome,^3^ respectively.

### Identification and Physicochemical Properties of KNOX Genes

A local BLASTp search was conducted using *A. thaliana* KNOX proteins as the query. Four conserved domains of KNOX: PF05920 (Homeobox KN domain), PF03790 (KNOX1 domain), PF03791 (KNOX2 domain), and PF03789 (ELK domain) were downloaded from the online database^4^ (Finn et al., 2010) to perform HMMER search (default parameters). Truncated and redundant proteins were manually removed after combining the BLASTp and HMMER results. NCBI Batch CD Search Tool^5^ was used for verifying the presence of the KNOX domain in candidate orchid KNOXs. The completed protein sequences of orchid KNOXs can be found in Supplementary Data Sheet. The physicochemical properties of KNOX genes were predicted by ExPASy database (Artimo et al., 2012). Subcellular localization was predicted by Plant-mPloc (Chou and Shen, 2010).

### Phylogenetic Analysis

The KNOX protein sequences of *A. thaliana*, *O. sativa*, *A. shenzhenica*, *P. equestris*, *C. ensifolium*, *C. goeringii*, and *D. chrysotoxum* were aligned with MAFFT (Rozewicki et al., 2019). The maximum likelihood (ML) method was adopted for constructing a phylogenetic tree using RAxML on the CIPRES Science Gateway web server (RAxML-HPC2 on XSEDE; Miller et al., 2015) under the Protein CAT model and GTR matrix with 1,000 bootstrap iterations. The output phylogenetic tree file was polished using Evolview (He et al., 2016).

### Motif and Gene Structure Analysis

GSDS^6^ (Hu et al., 2015) was used for analyzing KNOX gene structure. Conserved motifs in KNOX sequences were identified using MEME online tool^7^ (Bailey et al., 2009) with default parameters. Motif and gene structure of KNOX genes were visualized by TBtools (Chen et al., 2020).

### Protein Tertiary Structure Prediction

Protein tertiary structure prediction of orchid KNOXs was performed and visualized by SWISS-MODEL (Schwede et al., 2003).^8^ The tertiary structure was colored by rainbow order representing N to C terminus. The secondary structure was predicted by the SOPMA program^9^ (Geourjon and Deléage, 1995).

### Prediction of *Cis*-Acting Elements

A total of 2,000 bp upstream of all orchid KNOXs were extracted by TBtools (Chen et al., 2020). The online software PlantCARE (Lescot et al., 2002)^10^ was employed to identify and annotate the *cis*-acting elements found in the promoter regions. *Cis*-acting element numbers and responsive functions were visualized using TBtools (Chen et al., 2020).

### Collinearity and Selective Pressure

Given the chromosome-level genome assembly of *C. ensifolium*, *C. goeringii*, and *D. chrysotoxum*, genomic fasta files were merged pairwise to construct a database and query for BLASTp. The merged blast files and modified gff3 files of three species were examined using MCscanX (Wang et al., 2012) to identify the collinear blocks of KNOX genes between *D. chrysotoxum* and *C. goeringii*, *C. goeringii* and *C. ensifolium.* Dual_synteny_plotter tool of MCscanX (JCVI kit) was used to visualize the collinearity results. To assess the selection pressure of orchid KNOXs, gene pairs with similarities greater than 60% were identified by multiple sequence alignment using DNAman (Lynnon Corporation, Canada) with default parameters. Tbtools (Chen et al., 2020) was further used to calculate *K*a (non-synonymous substitutions per site), *K*s (synonymous substitutions per site), and *K*a/*K*s (evolutionary constraint) values.

### Expression Analysis

For transcriptomic analysis, RSEM (Li and Dewey, 2011) was used for transcript quantification and calculating the fragment per kilobase of transcript per million mapped reads (FPKM) value for each gene. Heatmaps using FPKM matrix were generated using tbtools (Chen et al., 2020).

To verify the expression pattern of KNOX genes, tender leaves, fully opened flowers, and mature pseudobulbs (stems) were sampled from *P. equestris*, *C. ensifolium*, *C. goeringii*, and *D. chrysotoxum* that were planted in Fujian Agriculture and Forestry University for RT-PCR analysis. Each tissue type was sampled in three replicates. Total RNA of these tissues was extracted using the RNAsimple Plant Kit. The RNA concentration for each tissue was in a range of 93–456 ng/μl with A260/280 value range from 1.92 to 2.15, indicating the extracted RNA is of high quality. Primer3Plus online tool^11^ was used to design specific PCR primers. Gene-specific primers for four selected genes and their corresponding internal reference genes are listed in Supplementary Table 1. Vazyme/R223 and Yeasen/11202ES03 kits were used for cDNA synthesis and qPCR, respectively. RT-qPCR was performed to verify the specific expression of Class I genes in the stem using *STM* homologs in orchids (*JL001208*, *GL17614*, *Peq022474*, *Maker101342*). All experiments were performed in three biological replicates with three technical replicates. The relative gene expression was calculated using the 2^–ΔΔ*CT*^ method.

### Gene Ontology Analysis of Orchid KNOXs

EggNOG-mapper v2^12^ was used to search against the eggNOG5.0 database (Huerta-Cepas et al., 2019) for gene ontology (GO) functional annotation. Orthology was predicted by sequence alignment, and bit-score or E-value setting was used to filter the low quality of orthology assignments; and functional classification was obtained based on the GO annotation terms associated with the proteins involved in known biological processes.

## Results

### Identification and Protein Features of Orchid KNOXs

A total of 32 putative KNOX genes (five in *A. shenzhenica*, six in *P. equestris*, five in *C. ensifolium*, nine in *C. goeringii*, and seven in *D. chrysotoxum*) were identified concerning four conserved domains characteristic of KNOX proteins, as previously detailed in the “Materials and Methods” section. These KNOX sequences varied considerably in the number of amino acids (aa), ranging from 85 to 928 with the molecular weight (MW) of which within a range of 9.81 to 102.35 kDa (Table 1). In addition, the deduced grand average of hydrophilic (GRAVY) values was all negative in five orchids’ KNOX proteins, suggestive of strong hydrophilicity. Except for four genes (*C. goeringii* (*GL09754*), *P. equestris* (*Peq027115*), *A. shenzhenica* (*Ash015432* and *Ash008116*)) that exhibited isoelectric points (pI) higher than eight, all other members were weakly acidic (ranging from 4.87 to 7.09). Most of them have the instability index (II) over 40, indicating that these proteins are unstable (Gasteiger et al., 2005). The results from subcellular location predictions showed that all KNOX proteins were located in nucleus, implying they may function on nucleus similar to most transcription factors (Table 1).

**TABLE 1:** Physicochemical properties and subcellular location of KNOX genes.

Gene ID	Name	AA^a^ (aa)	Mw^b^ (kDa)	pI^c^	II^d^	AI^e^	GRAVY^f^	Subcellular localization^g^
GL14512	GL14512	258	29.02	5.54	65.11	91.51	–0.353	Nucleus.
GL17195	GL17195	217	23.41	4.92	30.28	89.22	0.023	Nucleus.
GL17191	GL17191	138	15.99	5.77	36.87	63.7	–0.828	Nucleus.
GL17614	GL17614	299	33.35	5.85	46.02	76.42	–0.511	Nucleus.
GL09754	GL09754	143	16.78	8.61	47.25	73.71	–0.932	Nucleus.
GL21378	GL21378	321	36.35	5.9	52.47	77.2	–0.686	Nucleus.
GL26695	GL26695	162	18.85	6.09	47.18	63.27	–1.018	Nucleus.
GL33439	GL33439	339	38.46	5.45	56.08	73.75	–0.486	Nucleus.
GL13304	GL13304	359	40.61	6.73	37.5	69.86	–0.586	Nucleus.
Maker56383	Maker56383	309	34.58	5.95	45.64	61.26	–0.736	Nucleus.
Maker79872	Maker79872	310	35.21	5.83	69.83	84	–0.575	Nucleus.
Maker95458	Maker95458	335	36.87	4.87	43.16	68.54	–0.448	Nucleus.
Maker68614	Maker68614	287	32.13	4.99	37.87	60.87	–0.591	Nucleus.
Maker101342	Maker101342	301	33.43	5.85	44.92	76.91	–0.493	Nucleus.
Maker50091	Maker50091	348	38.82	5.57	56.52	77.18	–0.278	Nucleus.
Maker109170	Maker109170	162	18.93	7.07	38.93	60.86	–1.085	Nucleus.
JL019263	JL019263	302	34.3	6.01	67.98	85.93	–0.539	Nucleus.
JL005106	JL005106	381	42.53	7.09	50.05	74.65	–0.503	Nucleus.
JL001208	JL001208	299	33.35	5.85	46.02	76.42	–0.511	Nucleus.
JL017566	JL017566	202	22.74	7.01	47.99	81.68	–0.163	Nucleus.
JL023161	JL023161	85	9.81	6.74	45.06	70	–1.058	Nucleus.
JL001725	JL001725	332	37.24	6.04	41.73	63.49	–0.687	Nucleus.
Peq016118	Peq016118	313	35.72	6.46	61.17	81.05	–0.514	Nucleus.
Peq014500	Peq014500	320	35.93	5.57	49.82	69.81	–0.666	Nucleus.
Peq008060	Peq008060	352	39.69	6.01	55.02	72.67	–0.658	Nucleus.
Peq022474	Peq022474	301	33.61	6.09	49.21	73.62	–0.552	Nucleus.
Peq027115	Peq027115	240	27.27	8.97	65.02	83.79	–0.29	Nucleus.
Peq001326	Peq001326	324	36.56	6.05	45.04	65.68	–0.718	Nucleus.
Ashe008116	Ashe008116	286	32.98	9.36	79.09	73.32	–0.785	Nucleus.
Ashe015432	Ashe015432	928	102.35	9.25	47.91	76.95	–0.357	Nucleus.
Ashe012303	Ashe012303	279	30.97	5	36.75	71.79	–0.671	Nucleus.
Ashe017164	Ashe017164	314	34.55	5.85	48.06	68.47	–0.469	Nucleus.
Ashe004050	Ashe004050	362	40.62	5.56	49.21	61.52	–0.693	Nucleus.

*^a^Amino acid number.*

*^b^Molecular weight.*

*^c^Theoretical isoelectric point.*

*^d^Instability index.*

*^e^Aliphatic index.*

*^f^Grand average of hydrophobicity.*

*^g^Subcellular localization predicted by Plant-mPloc (Chou and Shen, 2010).*

### Phylogeny and Classification of KNOX Genes

**FIGURE 1:**
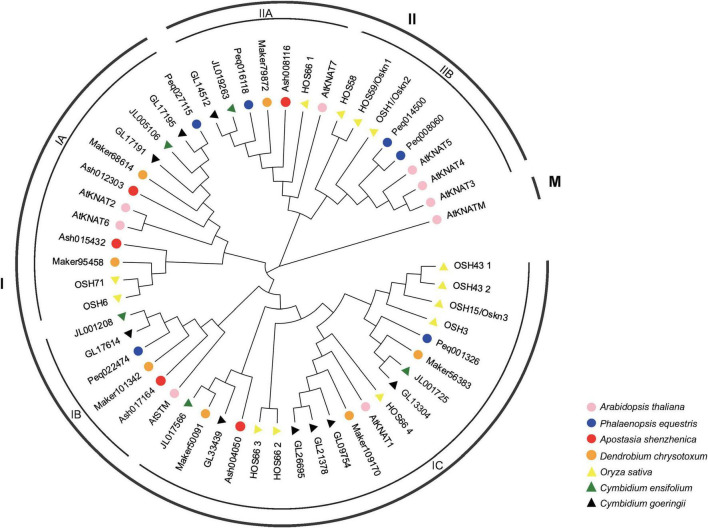
Phylogenetic tree of KNOX genes based on the KNOX protein sequences of seven plant species. The KNOX gene family was classified into three classes: Class I, Class II, and Class M with Class I divided into three subclades: IA, IB, and IC, and two subclades, IIA and IIB in Class II. KNOX protein sequences of all species are available in Supplementary Data Sheet.

The phylogenetic tree constructed by *A. thaliana*, *O. sativa*, *A. shenzhenica*, *P. equestris*, *C. ensifolium*, *C. goeringii*, and *D. chrysotoxum* has divided the KNOX genes into three clades, Class I, Class II, and Class III (Class M) (Figure 1), which is consistent with the studies have been done in *Arabidopsis*, rice, and *Populus* (Xiong et al., 2018). Among which, Class I was further divided into three subclades, designated as IA, IB, and IC, having eight, five, and 12 members in orchids, respectively. Class II comprises two subclades: IIA and IIB, including five and two members in orchids, respectively. No member of Class M (KNATM) was found in orchids, in accordance with a recent study that KNATM was exclusively found in eudicots (Jie et al., 2015).

### Collinearity and Evolutionary Analysis

**FIGURE 2:**
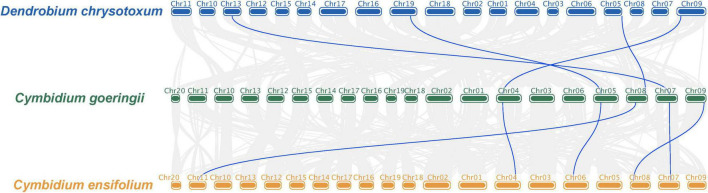
The collinearity of KNOX genes between *D. chrysotoxum* and *C. goeringii*, *C. goeringii* and *C. ensifolium.* The collinear analysis demonstrated a one-to-one correspondence among all KNOX genes in three orchids.

The collinear relationship among the KNOX genes of *D. chrysotoxum*, *C. goeringii*, and *C. ensifolium* was examined to find the potential events during KNOXs evolution in orchids. The collinear analysis demonstrated a one-to-one correspondence among all KNOX genes in three orchids, suggesting less reshuffling of KNOX orthologous and substantial genomic rearrangements after the lineages of *Dendrobium* and *Cymbidium* diverged (Figure 2). In addition, we examined the gene location on the chromosome for *C. goeringii* which contained the highest number of KNOX genes to look for the potential duplication event. The result showed that a small-scale tandem duplication might lead to the increasing members of KNOXs in *C. goeringii* (Supplementary Figure 1).

For selection pressure analysis, 38 gene pairs were selected based on sequence similarity for calculating the ratio of the number of non-synonymous substitutions per non-synonymous site (*K*a) to the number of synonymous substitutions per synonymous site (*K*s). The results showed that the *K*a/*K*s ratios of all KNOX genes were less than one, of which most values were below 0.4, indicating that all orchid KNOXs experienced strong purifying selection (Supplementary Table 2; Zhang et al., 2006).

### Motif and Gene Structure Analysis

**FIGURE 3:**
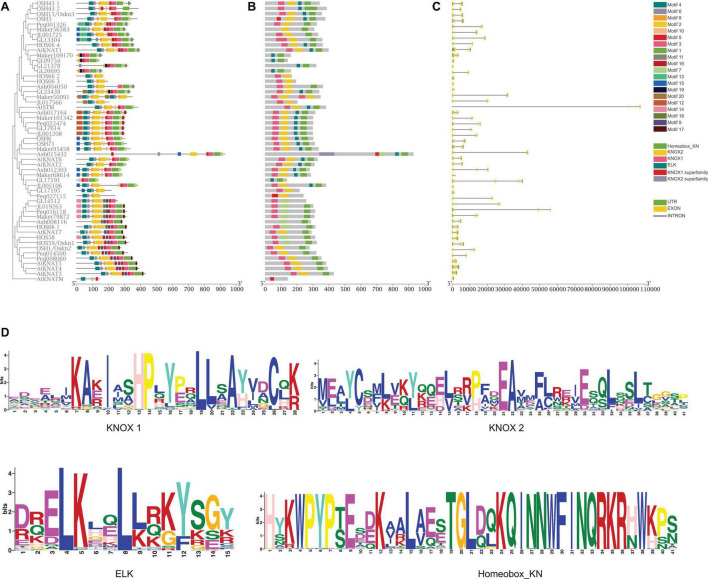
Gene structure, conserved motifs, conserved domains of KNOX genes. **(A)** Predicted motifs with the phylogenetic tree of orchid KNOXs. **(B)** Conserved KNOX domains. **(C)** Gene structure of KNOXs, with green blocks, yellow blocks, and gray lines representing upstream or downstream-untranslated regions (UTR), exons, and introns, respectively. **(D)** Sequence logo of Motif 4, motif 2, motif 5, and motif 1, which encoded the KNOX1, KNOX2, ELK, and Homeobox domains, respectively.

Motifs of KNOX proteins in *A. thaliana*, rice, and five orchids were examined using the online analysis tool MEME, and 20 motifs were set as upper bound (Figure 3A). The number of KNOX motifs ranged from three (*AtKNATM*) to 14 (*GL13304*). Motif 4, motif 2, motif 5, and motif 1 encoded the KNOX1, KNOX2, ELK, and Homeobox domains, respectively. Although most orchid KNOX proteins contained these four conserved protein domains (Figures 3B,D), motif structure differs in each subclade. For instance, motifs 9, 14, and 17 were only present in Class II. Motif 19 was specifically distributed in *C. goeringii* and *D. chrysotoxum* and motif 20 was only present in *D. chrysotoxum*, *C. goeringii*, and *C. ensifolium*.

To further explore the characteristics of KNOX genes in orchids, intron–exon structure was analyzed (Figure 3C). The results showed that the orchid KNOX family is composed of 1–9 exons and 1–4 introns. Although the similarity in gene structure was found in each subclade, orchid KNOX genes have a high degree of variance in intron length and exon numbers in comparison with *A. thaliana* and rice. In general, most Class I genes exhibited longer intron length than Class II genes. Notably, in Class IB, orchid KNOX genes displayed a significant shortening intron length than *AtSTM*, whereas in other clades, most orchid KNOXs have longer intron than *A. thaliana* and rice (Figure 3C), which might be a unique feature of Orchidaceae.

### Protein Tertiary Structure Prediction

**FIGURE 4:**
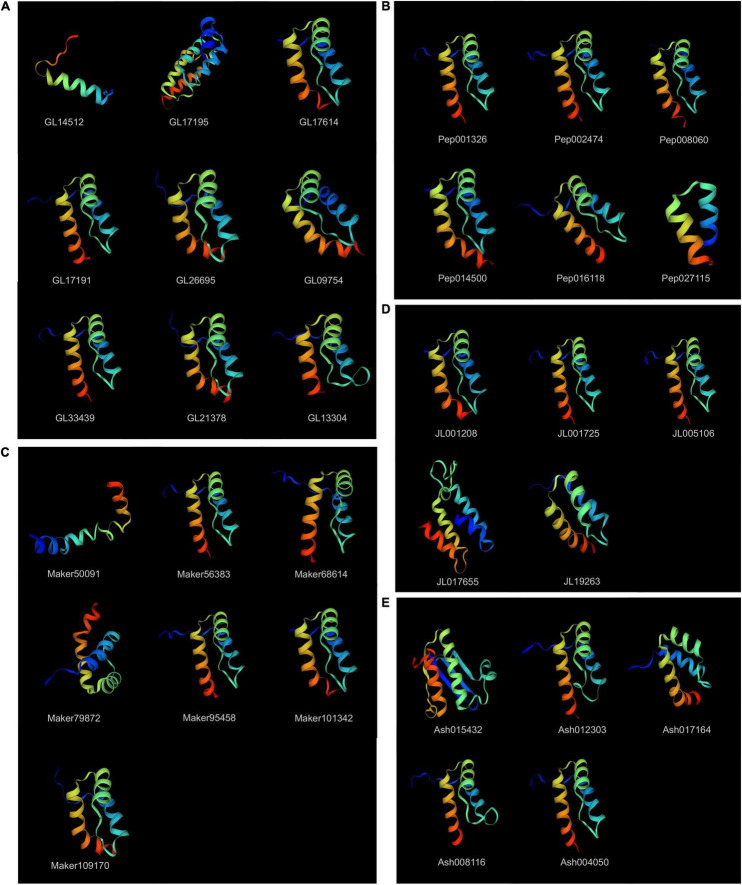
Protein tertiary structure of KNOX genes. **(A)** *C. goeringii*. **(B)** *P. equestris.* **(C)** *D. chrysotoxum.* **(D)** *C. ensifolium*. **(E)** *A. shenzhenica.* The tertiary structures were colored by rainbow order, representing N to C terminus.

The tertiary structures of most KNOXs in orchids were highly conserved, characterized by three helices, among which helices I and II were connected by a loop structure, helices II and helices III formed a helix–turn–helix motif (Figure 4). With the exception, *GL14512* (Class IIA) showed a merged helix without loop or turn, and the other two helices *GL17195* (Class IA) and *JL017655* (Class IC) had four helices, and *Maker50091* (Class IC) exhibited very short three helices. The secondary structure prediction indicated that all orchid KNOX proteins composed of α-helix (Hh), random coils (Cc), extended strands (Ed), and β-turns (Tt), the mean of which account for 48.00%, 7.02%, 4.98%, and 40.00% of the protein structure, respectively (Supplementary Table 3).

### *Cis*-Acting Regulatory Elements Analysis

**FIGURE 5:**
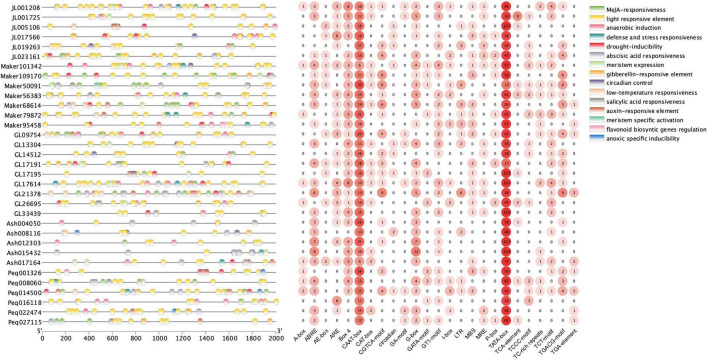
*Cis*-acting elements in the promoter regions of KNOX genes. Elements with similar regulatory functions are displayed in the same color. Numbers of each type of element are shown on the right side.

To investigate the regulatory functions of KNOXs, the 2,000 bp promoter regions of orchid KNOX genes were retrieved for the identification of putative *cis*-elements. A total of 797 *cis*-acting elements attributing to 25 types and 15 responsive functions were identified (Figure 5 and Supplementary Table 4). Among these elements, TATA-box made up the most common elements (62.62%), followed by CAAT-box (15.04%) (Supplementary Table 5). *Cis*-element functions included phytohormone responsiveness for gibberellin, auxin, methyl jasmonate (MeJA), salicylic acid, and abscisic acid (ABA); stress responsiveness such as drought, anoxic, anaerobic, low-temperature; and growth and development elements as light response and circadian control (Figure 5). Each KNOX gene contained multiple types of elements with light responsiveness as the most occurring element function, supporting the key roles of light that mediate KNOX function during plant development (Figure 5). MeJA-responsive element, followed by ABA-responsiveness, constituting the second and third most abundant type (Supplementary Table 4), respectively, were also widely distributed in most orchid KNOXs, implying they may exert function in modulating these two phytohormones. In particular, we also found 14 elements that are related to meristem expression and meristem-specific activation, which is consistent with the instrumental roles of KNOX in the maintenance of meristem homeostasis.

### Expression Analysis

**FIGURE 6:**
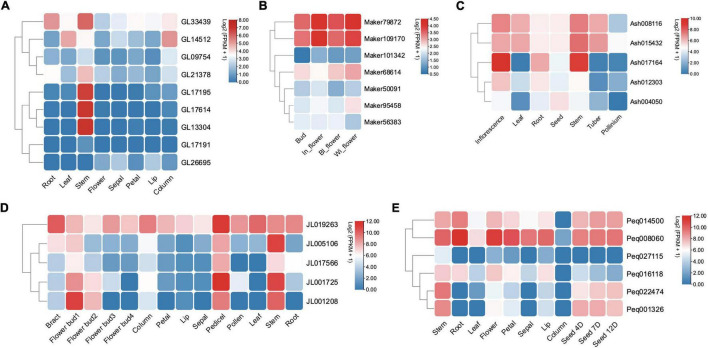
The expression profile of KNOX genes among different tissues in five orchids. **(A)** *C. goeringii.* **(B)** *D. chrysotoxum.* In_flower, initial opened flower; BI_flower, fully opened flower; Wi_flower, withered flower. **(C)** *A. shenzhenica.* **(D)** *C. ensifolium.* Flower bud1, flower bud at an early stage; Flower bud2, flower bud at the middle stage; Flower bud3, flower bud at a late stage. **(E)** *P. equestris.* Seed 4D, 4 day’s seed; Seed 7D, 7 day’s seed; Seed 12D, 12 day’s seed.

Expression analysis was conducted based on five orchids’ transcriptome data of various tissues including different flower segments, leaves, pseudobulbs (stem), root, and seed. The expression profile showed that Class II KNOX genes were expressed broadly in differentiating tissues and mature organs, while the expression of Class I genes was more confined to less differentiated tissues with SAM-like pseudobulbs (stem) (Figure 6). For instance, in *C. goeringii*, Class I genes such as *GL21378*, *GL33439*, *GL17614*, and *GL17195* exhibited an exclusive expression in the stem (Figure 6A). In addition, *Ash017164*, *JL001208*, and *Peq022474*, which were homologs to *STM* have high expression levels in stem, inflorescence, pedicels, and seeds that bear numerous meristematic regions, indicating the Class I KNOX genes’ function in meristem maintenance.

**FIGURE 7:**
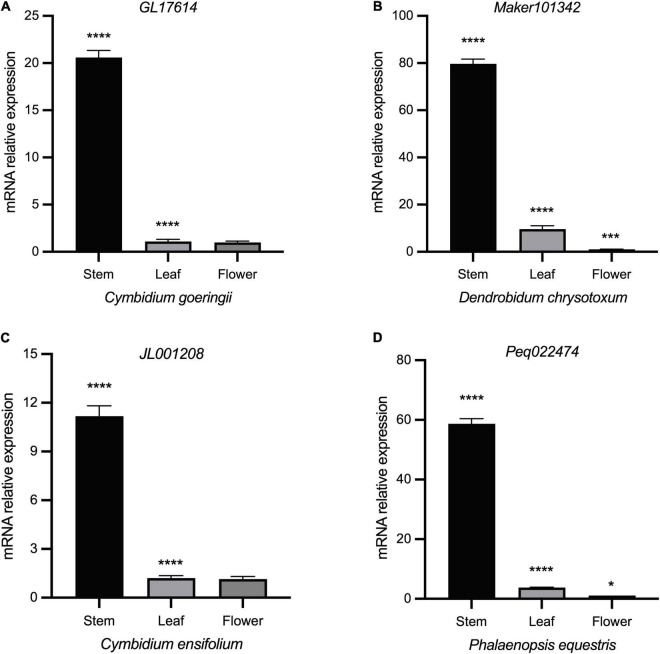
Expression profiles of different tissues of STM homologs in four orchids by RT-qPCR. **(A)** *C. goeringii.* **(B)** *D. chrysotoxum.* **(C)** *C. ensifolium.* **(D)** *P. equestris.* ANOVA multiple comparisons were performed with star marks *, ***, and **** representing adjusted *p* < 0.05, *p* < 0.001, and *p* < 0.0001, respectively (Supplementary Table 6).

Whereas Class II genes such as *JL019263* and *Peq008060* were highly expressed in almost all vegetative and reproductive tissues (Figures 6B–E), demonstrating a widespread expression and their possible role in promoting tissue differentiation. *STM* has an early origin compared to other members of Class I (Furumizu et al., 2015; Frangedakis et al., 2017), and has been shown to positively regulate *KNAT1* and *KNAT2*, with *KNAT6* performing redundant function with *STM* in SAM maintenance (Scofield et al., 2014; Bueno et al., 2020). To further investigate the specific roles of Class I KNOX genes, gene expression of *STM* homologs in *C. goeringii*, *P. equestris*, *C. ensifolium*, and *D. chrysotoxum* was analyzed in their leaves, flowers, and stems by RT-qPCR (Figure 7 and Supplementary Table 1). In all examined orchids, the *STM* homologs showed extremely high expression in stems but were barely detected in flowers and leaves, further verifying that Class I genes have a tissue-specific expression in the stem. As an early diverging Class I KNOX member, the high expression of *STM* could also explain the concomitant elevated expression of other Class I members in the stem (Figure 6). A critical next step will be the functional analysis of these two classes to unravel the underlying roles of KNOXs in orchids.

### Gene Ontology Analysis of Orchid KNOXs

**FIGURE 8:**
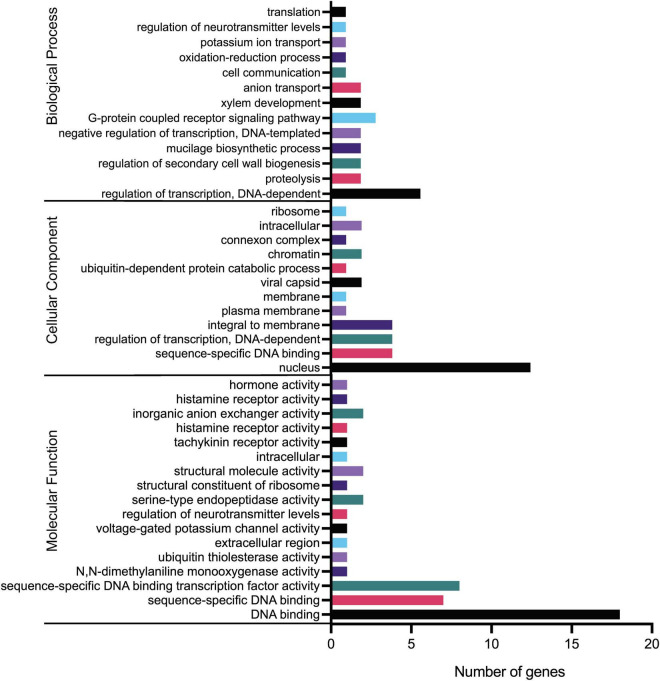
Gene ontology (GO) terms of orchid KNOX genes. Annotation details for each gene are listed in Supplementary Table 7.

Gene ontology analysis was performed to delineate gene functional classifications of orchid KNOXs and investigate the important biological processes they might be involved in. As a result, GO terms “regulation of transcription,” “nucleus,” and “DNA binding” constitute the greatest number of genes for GO ontologies “Biological Process,” “Cellular Component,” and “Molecular Function,” respectively (Figure 8 and Supplementary Table 7). The results match the fact that KNOX is a premier regulator that is associated with numerous downstream transcriptional networks and functions mostly in the nucleus (Table 1). Several other terms such as “regulation of secondary cell wall biogenesis” and “hormone activity” are also consistent with the KNOX’s function reported in previous studies (Tabata et al., 2010; Wang et al., 2020).

## Discussion

The primary architecture of plants derives from the SAM, producing leaves, internodes, and axillary buds. As the key regulator functioning in the maintenance of meristematic potentials, KNOX gene family is closely linked to lateral organ morphogenesis. In this study, we identified 32 KNOX genes in five orchids and classified them into two classes: Class I and Class II based on phylogenetic relationship, with no Class M member has been identified, owning to this clade is exclusive to some eudicot species (Jie et al., 2015; Bueno et al., 2020). Class IC, represented by *Arabidopsis* genes *KNAT1*, has the greatest number of orchid KNOX homologs, in which *D. chrysotoxum*, *C. ensifolium*, and *C. goeringii* contained more than one member (Figure 1). Evolutionary novelty is thought to be driven by gene duplication, which is rife in angiosperms (Van de Peer et al., 2017). A study based on 48 species revealed that the number of KNOX genes among angiosperms spanning from four to 28, while the duplicated homologs to *KNAT1* clade were only observed in *Gmax glyma* (dicots) and *O. sativa* (monocots) (Jie et al., 2015). Orchids exhibited a small number of KNOX genes (5–9), the genus-specific duplication in Class IC, therefore, may underlie the possible neo-functionalization of their roles in contributing to important innovations of plant architecture in Orchidaceae.

Variation in the gene structure of a gene family among different species has been largely uncovered thanks to the development of whole-genome sequencing. Given the conserved nature of gene structure within the same clade, we found that orchid KNOXs have a similar exon–intron structure compared to *A. thaliana* and *O. sativa*, except that most orchids exhibited longer intron length (Figure 3C). Long intron has been reported in almost all sequenced orchid species (Cai et al., 2014; Zhang et al., 2016, 2017; Yuan et al., 2018; Ai et al., 2021), which might be a unique feature of Orchidaceae. Longer introns are favored in the course of gene evolution because they promote the efficiency of natural selection by increasing the recombination between two adjacent exons (Jo and Choi, 2015), which may account for the marvelous richness of orchids.

Gene expression at the promoter region is primarily regulated by the *cis*-acting elements situated upstream of the transcriptional start site (Hernandez-Garcia and Finer, 2014). In this study, we identified diverse function types of regulatory elements in orchid KNOXs’ promoter regions, which were further categorized as phytohormone responsive, stress-responsive, and growth and development elements (Figure 5). Our results showed that a large proportion of *cis*-elements are acting in light responsiveness, indicative of environmental cues such as light could exert an impact on KNOX’s function. In addition, many elements are responsive to JA, GA, ABA, and auxin. Indeed, KNOXs have important functions in modulating these phytohormones. It has been reported that auxin response factor genes positively regulate JA biosynthesis in floral organs *via* the suppression of Class 1 *KNOX* genes (Tabata et al., 2010). In leaf, *KNOX* genes are repressed to maintain a low cytokinin (CK) to high gibberellin (GA) ratio, thus switching SAM from an indeterminate state to leaf-specific growth (Ichihashi and Tsukaya, 2015). Meanwhile, Class II KNOX genes play a crucial role in regulating ABA signaling during organ development (Jia et al., 2021b). The diverse functions of *cis*-elements reflect the multiple roles of KNOX genes involved in plant growth and development.

Intra-genomic comparisons between *D. chrysotoxum*, *C. goeringii*, and *C. ensifolium’s* chromosomes showed that their KNOX genes were in a one-to-one correspondence, supporting no duplication events have occurred in orchid KNOXs (Figure 2). Moreover, *C. goeringii* and *C. ensifolium* have a nearly one-to-one syntenic relationship between their chromosomes, demonstrating there were no obvious inter-chromosomal structure variations after the two species diverged. The *K*a/*K*s value indicated that all orchid KNOXs undergo strong purifying selection (Supplementary Table 2), which is critical to eliminating newly arising deleterious mutations thus maintaining biological function (Cvijović et al., 2018), indicating the conservation of this gene family has been reinforced across the Orchidaceae lineage.

Neo-functionalization of transcription factors can be achieved through changes in expression pattern or function alteration (Furumizu et al., 2015). In angiosperms, Class I and II KNOX genes play contrary roles in plant growth and development, with Class I and Class II members functioning in meristems maintenance and differentiation of all aerial organs, respectively. It is clear from our study that Class II KNOX genes have a widespread expression in differentiated tissues, while the expression of Class I genes was more restricted to the stem that contains many meristematic regions (Figures 6, 7). It is suggested that the diversified expression pattern of Class II genes may be due to the neofunctionalization during the gene duplication event of an ancestral KNOX gene (Furumizu et al., 2015). The opposing activities between Class I and Class II KNOX genes may underlie the molecular mechanism of key innovations and modification of plant architecture *via* elaboration of transcriptional networks. Characterization of the loss/gain-of-function mutants and physiological experiments on overexpressed phenotypes may illuminate more undetected roles of Class II KNOX genes.

## Conclusion

Members of the KNOX gene family are versatile effectors regulating SAM maintenance and hormonal signaling thus influencing many facets of plant growth and development. A total of 32 orchid KNOX genes were identified and assigned to two subclasses by phylogenetic analysis with more members found in Class I. Motif, gene structure, and protein tertiary prediction suggested that orchid KNOXs were conserved among different species and a small scale tandem duplication gave rise to more members in *C. goeringii*. We showed that the specific functions of *cis*-element in meristem expression and activation are acting in concert with the pivotal role of KNOXs in SAM. In addition, the expression patterns generated by transcriptomic and RT-qPCR data supported a tissue-specific expression of Class I genes in the stem where a pool of pluripotent stem cells was generated. Our study presents a comprehensive analysis for uncovering the function and expression pattern of KNOX genes in Orchidaceae. These results build a foundation for further understanding of how KNOX genes have been co-opted in regulating various forms of lateral organs and shed light on the plasticity of plant architecture. A critical next step will be the functional analysis of KNOX in non-model plants to characterize the additional role of KNOX in the context of land plant evolution.

## Data Availability Statement

The original contributions presented in the study are included in the article/Supplementary Material, further inquiries can be directed to the corresponding authors.

## Author Contributions

W-LY and Z-JL conceived and designed the research. DZ and SL performed the data analysis and wrote the manuscript. All authors contributed to the article and approved the submitted version.

## Conflict of Interest

The authors declare that the research was conducted in the absence of any commercial or financial relationships that could be construed as a potential conflict of interest.

## Publisher’s Note

All claims expressed in this article are solely those of the authors and do not necessarily represent those of their affiliated organizations, or those of the publisher, the editors and the reviewers. Any product that may be evaluated in this article, or claim that may be made by its manufacturer, is not guaranteed or endorsed by the publisher.

## Footnotes

1
https://bigd.big.ac.cn/

2
http://www.arabidopsis.org

3
https://phytozome-next.jgi.doe.gov/

4
http://pfam.xfam.org/

5
https://www.ncbi.nlm.nih.gov/Structure/bwrpsb/bwrpsb.cgi

6
http://gsds.gao-lab.org/

7
http://meme-suite.org/tools/meme

8
https://swissmodel.expasy.org/interactive

9
https://npsa-prabi.ibcp.fr/

10
http://bioinformatics.psb.ugent.be/webtools/plantcare/html/

11
http://www.primer3plus.com/cgi-bin/dev/primer3plus.cgi

12
http://eggnog-mapper.embl.de/
