# Supplementary material for: Genome-Wide Identification and Expression Pattern Analysis of KNOX Gene Family in Orchidaceae
Source: Front Plant Sci. 2022 May 27;13:901089. doi: 10.3389/fpls.2022.901089 (PMC9197187; doi:10.3389/fpls.2022.901089)
Supplement: Supplementary file 2 [file Data_Sheet_2.docx]

**Supplementary Information**

**Genome-wide identification and expression pattern analysis of KNOX gene family in Orchidaceae**

Diyang Zhang^1^, Siren Lan^1^, Wei-Lun Yin^1, 2†^, Zhong-Jian Liu^1†^

**Content**

[Supplementary Figures 3](#_Toc101045107)

[Supplementary Figure 1. Gene location at chromosomes for *C. goeringii*. 3](#_Toc101045108)

[Supplementary Tables 4](#_Toc101045109)

[Supplementary Table 1. The primers of *STM* in four orchids and their internal reference genes. 4](#_Toc101045110)

[Supplementary Table 2. *K*a/*K*s value of gene pairs. 5](#_Toc101045111)

[Supplementary Table 3. Secondary structure prediction of orchid KNOX protein sequence. 6](#_Toc101045112)

[Supplementary Table 4. *Cis*-acting element identified in the promotor region (see separate file). 8](#_Toc101045113)

[Supplementary Table 5. *Cis*-acting element types and numbers (see separate file). 8](#_Toc101045114)

[Supplementary Table 6. Statistical analysis using ANNOVA multiple comparisons for qPCR expression levels between each tissue. 9](#_Toc101045115)

[Supplementary Table 7. Gene ontology (GO) annotation of orchid KNOX genes (see separate file). 10](#_Toc101045116)

# Supplementary Figures


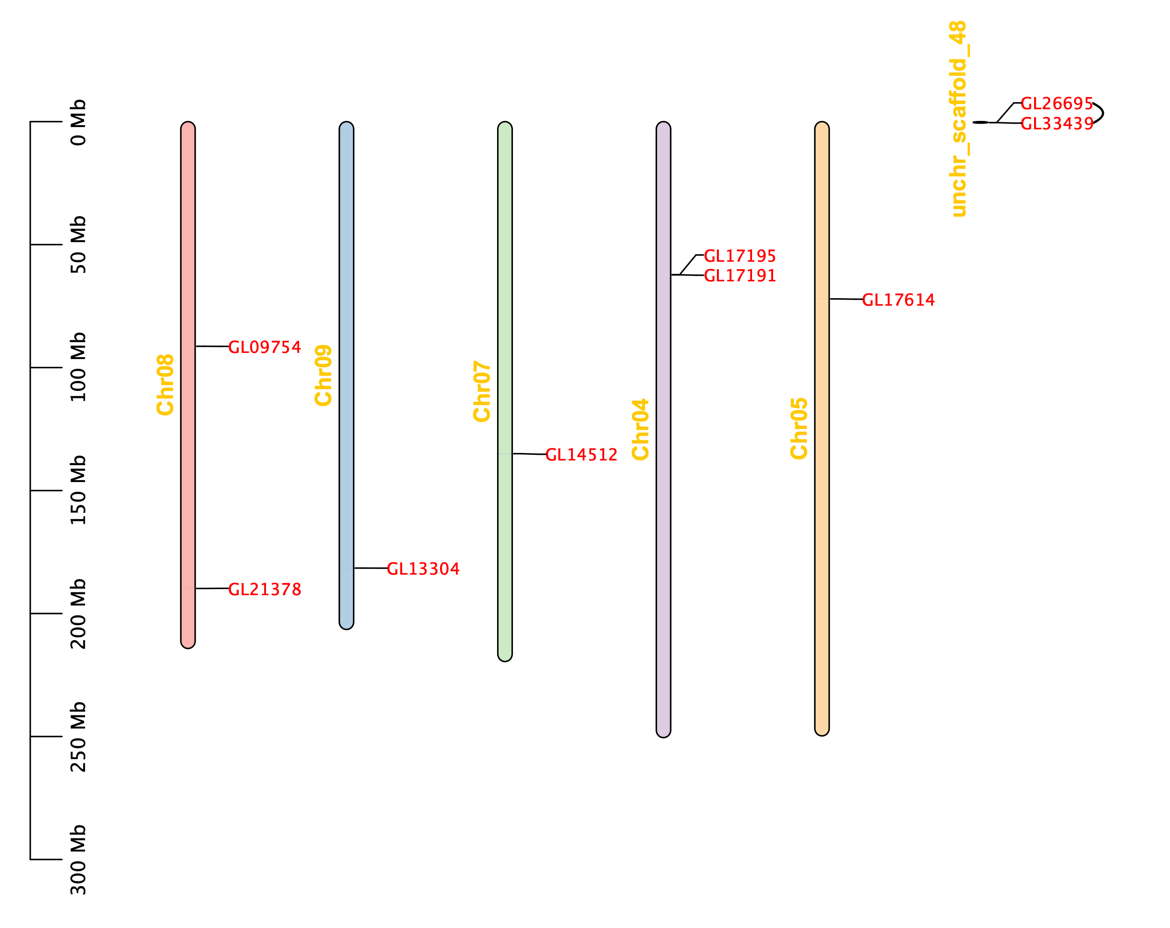


## Supplementary Figure 1. Gene location at chromosomes for *C. goeringii*.

# Supplementary Tables

## Supplementary Table 1. The primers of *STM* in four orchids and their internal reference genes.

| **Genes** | **(5’–3’) Forward primer** | **(5’–3’) Reverse primer** |
| --- | --- | --- |
| *JL013805* (internal reference) | CTGAAGCCCCTCTCAATCCC | CTGAAGCCCCTCTCAATCCC |
| *JL001208* | GGTGGACTCGCCACTACAAA | CGAGACCACCGTCCATGAAA |
| *GL17958* (internal reference) | ATATGCTAGTGGCCGCACAA | TTCCCGTTCCGCAGTAGTTG |
| *GL17614* | AAAACGGTTCGTCCGAGGAT | TTTGTAGTGGCGAGTCCACC |
| *Peq023339* (internal reference) | ATCACAATAGGGGCTGAGCG | TCAGCAATGCCAGGGAACAT |
| *Peq022474* | TTGCCAGGCTAGAAGAAGCC | AGGTGGCAAAGAAAGCGAGA |
| *Maker75111*(internal reference) | CAGGCATCCATGAGACGACA | ACTTCCTCTCTGGTGGAGCTA |
| *Maker101342* | CGGTTCGTCTGAGGATGACA | GTCAAGCAGTTGTTGCCTGG |

## Supplementary Table 2. *K*a/*K*s value of gene pairs.

| **ID** | **Gene name** | ***K*a** | ***K*s** | ***K*a/*K*s** |
| --- | --- | --- | --- | --- |
| Peq014500 | Peq008060 | 0.099389 | 2.911331 | 0.034139 |
| Peq016118 | Peq008060 | 0.298922 | 6.658559 | 0.044893 |
| Maker109170 | Maker56383 | 0.215245 | 3.524508 | 0.061071 |
| Ash015432 | Ash004050 | 0.529517 | 6.560871 | 0.080708 |
| GL26695 | GL13304 | 0.194633 | 2.135219 | 0.091154 |
| GL09754 | GL13304 | 0.22387 | 2.064604 | 0.108432 |
| JL001725 | JL017566 | 0.469382 | 4.187456 | 0.112092 |
| Maker101342 | Maker56383 | 0.397726 | 3.380392 | 0.117657 |
| Maker95458 | Maker56383 | 0.531241 | 3.973738 | 0.133688 |
| Ash015432 | Ash012303 | 0.362123 | 2.453127 | 0.147617 |
| Maker79872 | Maker101342 | 0.786538 | 5.05139 | 0.155707 |
| Ash012303 | Ash004050 | 0.429004 | 2.656591 | 0.161487 |
| GL17614 | GL21378 | 0.431134 | 2.519981 | 0.171086 |
| Maker101342 | Maker68614 | 0.433985 | 2.530969 | 0.17147 |
| JL001725 | JL001208 | 0.366817 | 2.132712 | 0.171996 |
| Maker79872 | Maker109170 | 0.622826 | 2.854997 | 0.218153 |
| GL21378 | GL13304 | 0.460366 | 2.013696 | 0.228618 |
| Maker101342 | Maker50091 | 0.704689 | 3.038337 | 0.231932 |
| Ash017164 | Ash004050 | 0.382126 | 1.436425 | 0.266026 |
| Ash012303 | Ash017164 | 0.399429 | 1.476229 | 0.270574 |
| Maker50091 | Maker56383 | 0.513743 | 1.834578 | 0.280034 |
| Ash008116 | Ash004050 | 0.798731 | 2.842651 | 0.280981 |
| Maker79872 | Maker50091 | 0.906007 | 2.726456 | 0.332302 |
| Peq022474 | Peq027115 | 0.76477 | 2.237757 | 0.341758 |
| Peq016118 | Peq027115 | 1.036744 | 2.828073 | 0.36659 |
| Maker79872 | Maker95458 | 0.797967 | 2.044028 | 0.390389 |
| Ash008116 | Ash012303 | 0.733044 | 1.8293 | 0.400724 |
| JL001208 | JL017566 | 0.679706 | 1.680251 | 0.404527 |
| Peq008060 | Peq027115 | 1.041879 | 2.136474 | 0.487663 |
| GL21378 | GL33439 | 0.337214 | 0.685662 | 0.491807 |
| GL26695 | GL33439 | 0.01566 | 0.030985 | 0.505384 |
| GL09754 | GL21378 | 0.060777 | 0.098807 | 0.615114 |
| GL09754 | GL33439 | 0.084814 | 0.105875 | 0.80108 |
| GL09754 | GL26695 | 0.062776 | 0.067186 | 0.934353 |

## Supplementary Table 3. Secondary structure prediction of orchid KNOX protein sequence.

|  | **Alpha helix (Hh)** | **310 helix (Gg)** | **Pi helix (Ii)** | **Beta bridge (Bb)** | **Extended strand (Ee)** | **Beta turn (Tt)** | **Bend region (Ss)** | **Random coil (Cc)** | **Ambiguous states** | **Other states** |
| --- | --- | --- | --- | --- | --- | --- | --- | --- | --- | --- |
| Ash008116 | 44.41% | 0% | 0% | 0% | 3.15% | 2.45% | 0% | 50% | 0% | 0% |
| Ash015432 | 40.52% | 0% | 0% | 0% | 15.62% | 7.76% | 0% | 36.10% | 0% | 0% |
| Ash012303 | 48.39% | 0% | 0% | 0% | 3.23% | 4.66% | 0% | 43.73% | 0% | 0% |
| Ash017164 | 42.04% | 0% | 0% | 0% | 6.69% | 3.83% | 0% | 47.45% | 0% | 0% |
| Ash004050 | 44.20% | 0% | 0% | 0% | 6.08% | 5.52% | 0% | 44.20% | 0% | 0% |
| JL001725 | 46.99% | 0% | 0% | 0% | 5.12% | 6.93% | 0% | 40.96% | 0% | 0% |
| JL019263 | 59.27% | 0% | 0% | 0% | 0.99% | 2.32% | 0% | 37.42% | 0% | 0% |
| JL005106 | 41.47% | 0% | 0% | 0% | 11.81% | 6.30% | 0% | 40.42% | 0% | 0% |
| JL001208 | 46.82% | 0% | 0% | 0% | 6.02% | 4.35% | 0% | 42.81% | 0% | 0% |
| JL017566 | 51.98% | 0% | 0% | 0% | 10.89% | 4.46% | 0% | 32.67% | 0% | 0% |
| Peq016118 | 53.99% | 0% | 0% | 0% | 5.43% | 2.24% | 0% | 38.34% | 0% | 0% |
| Peq014500 | 51.25% | 0% | 0% | 0% | 6.88% | 4.38% | 0% | 37.50% | 0% | 0% |
| Peq008060 | 51.42% | 0% | 0% | 0% | 4.26% | 2.27% | 0% | 42.05% | 0% | 0% |
| Peq022474 | 43.19% | 0% | 0% | 0% | 3.65% | 4.65% | 0% | 48.50% | 0% | 0% |
| Peq027115 | 51.25% | 0% | 0% | 0% | 7.50% | 4.58% | 0% | 36.67% | 0% | 0% |
| Peq001326 | 42.90% | 0% | 0% | 0% | 9.26% | 6.16% | 0% | 41.67% | 0% | 0% |
| Maker79872 | 59.68% | 0% | 0% | 0% | 2.58% | 1.61% | 0% | 36.13% | 0% | 0% |
| Maker95458 | 49.86% | 0% | 0% | 0% | 4.48% | 5.97% | 0% | 39.70% | 0% | 0% |
| Maker68614 | 52.96% | 0% | 0% | 0% | 3.83% | 5.23% | 0% | 37.98% | 0% | 0% |
| Maker101342 | 50.50% | 0% | 0% | 0% | 3.99% | 4.32% | 0% | 41.20% | 0% | 0% |
| Maker50091 | 50.86% | 0% | 0% | 0% | 13.51% | 5.17% | 0% | 30.46% | 0% | 0% |
| Maker109170 | 37.65% | 0% | 0% | 0% | 11.11% | 8.02% | 0% | 43.21% | 0% | 0% |
| Maker56383 | 44.34% | 0% | 0% | 0% | 6.15% | 5.50% | 0% | 44.01% | 0% | 0% |
| GL14512 | 62.79% | 0% | 0% | 0% | 2.71% | 1.16% | 0% | 33.33% | 0% | 0% |
| GL17195 | 57.60% | 0% | 0% | 0% | 5.53% | 5.53% | 0% | 31.34% | 0% | 0% |
| GL17191 | 49.28% | 0% | 0% | 0% | 12.32% | 9.42% | 0% | 28.99% | 0% | 0% |
| GL17614 | 48.49% | 0% | 0% | 0% | 5.02% | 4.68% | 0% | 41.81% | 0% | 0% |
| GL09754 | 48.95% | 0% | 0% | 0% | 6.29% | 6.99% | 0% | 37.76% | 0% | 0% |
| GL21378 | 33.33% | 0% | 0% | 0% | 15.26% | 7.48% | 0% | 43.93% | 0% | 0% |
| GL26695 | 36.42% | 0% | 0% | 0% | 11.73% | 4.94% | 0% | 46.91% | 0% | 0% |
| GL33439 | 48.97% | 0% | 0% | 0% | 7.08% | 5.60% | 0% | 38.35% | 0% | 0% |
| GL13304 | 44.29% | 0% | 0% | 0% | 6.41% | 4.74% | 0% | 44.57% | 0% | 0% |
| Average | 48.00% | 0% | 0% | 0% | 7.02% | 4.98% | 0% | 40.00% | 0% | 0% |

## Supplementary Table 4. *Cis*-acting element identified in the promotor region (see separate file).

## Supplementary Table 5. *Cis*-acting element types and numbers (see separate file).

## Supplementary Table 6. Statistical analysis using ANNOVA multiple comparisons for qPCR expression levels between each tissue.

| **Species** | **Tukey's multiple comparisons test** | **Mean Diff.** | **95.00% CI of diff.** | **Below threshold?** | **Summary** | **Adjusted P value** |
| --- | --- | --- | --- | --- | --- | --- |
| *D. chrysotoxum* | Stem vs. Leaf | 70 | 66.4 to 73.6 | Yes | **** | <0.0001 |
|  | Stem vs. Flower | 78.6 | 75.0 to 82.2 | Yes | **** | <0.0001 |
|  | Leaf vs. Flower | 8.61 | 5.04 to 12.2 | Yes | *** | 0.0008 |
| *C. goeringii* | Stem vs. Leaf | 18.6 | 17.5 to 19.7 | Yes | **** | <0.0001 |
|  | Stem vs. Flower | 19.6 | 18.5 to 20.7 | Yes | **** | <0.0001 |
|  | Leaf vs. Flower | 0.995 | -0.0974 to 2.09 | No | ns | 0.0703 |
| *C. ensifolium* | Stem vs. Leaf | 9.95 | 9.03 to 10.9 | Yes | **** | <0.0001 |
|  | Stem vs. Flower | 10.2 | 9.27 to 11.1 | Yes | **** | <0.0001 |
|  | Leaf vs. Flower | 0.233 | -0.679 to 1.15 | No | ns | 0.7251 |
| *P. equestris* | Stem vs. Leaf | 54.9 | 52.5 to 57.4 | Yes | **** | <0.0001 |
|  | Stem vs. Flower | 57.7 | 55.2 to 60.1 | Yes | **** | <0.0001 |
|  | Leaf vs. Flower | 2.76 | 0.299 to 5.21 | Yes | * | 0.0318 |

## Supplementary Table 7. Gene ontology (GO) annotation of orchid KNOX genes (see separate file).
